# Does Predation Influence the Seasonal and Diel Timing of Moose Calving in Central Ontario, Canada?

**DOI:** 10.1371/journal.pone.0150730

**Published:** 2016-04-15

**Authors:** Brent R. Patterson, Kenneth J. Mills, Kevin R. Middel, John F. Benson, Martyn E. Obbard

**Affiliations:** 1 Ontario Ministry of Natural Resources and Forestry, Wildlife Research and Monitoring Section, Trent University, Peterborough, Ontario, Canada; 2 Environmental and Life Sciences Graduate Program, Trent University, Peterborough, Ontario, Canada; University of Missouri, UNITED STATES

## Abstract

Birth synchrony is well documented among ungulates and is hypothesised to maximize neonate survival, either by minimizing the risk of predation through predator swamping or by synchronising birthing with increased seasonal food availability. We used encapsulated vaginal implant transmitters to locate and capture neonatal moose calves and document the seasonal and diel timing of parturition in two adjacent study areas with different predation pressure in central Ontario, Canada. We tested the hypothesis that predation promotes earlier and more synchronous birth of moose calves. Across both areas, proportionately more births occurred during the afternoon and fewer than expected occurred overnight. Mean date of calving averaged 1.5 days earlier and calving was also more synchronous in the study area with heavier predation pressure, despite average green-up date and peak Normalized Difference Vegetation Index date occurring 2 days later in this study area than in the area receiving lighter predation pressure. We encourage analysis of data on timing of parturition from additional study areas experiencing varying degrees of predation pressure to better clarify the influence of predation in driving seasonal and diel timing of parturition in temperate ungulates.

## Introduction

Birth synchrony is well documented among ungulates [1, 2, 3, 4, 5] and is generally hypothesised to maximize neonate survival, either by minimizing the risk of predation through predator swamping [6, 7, 8, 9] or by synchronising birthing with increased seasonal food availability [10, 11, 12]. The timing of parturition, independent of synchrony, may also influence the survival of neonates. Early-born neonates may experience reduced predation relative to later born individuals by achieving greater size and reduced vulnerability to predators by the time predators recognize the birth pulse and begin to focus on the present year’s cohort of neonates [13, 14]. Alternatively, Keech et al. [14] provided evidence that moose timed parturition to maximize the use of the growing season, as previously suggested by Bowyer et al. [14]. These authors suggested that moose time parturition as early as possible following winter to maximize the growing season for calves and speculated that young moose born too late in spring would have insufficient time to accrue body reserves necessary to survive harsh conditions in winter [10, 14]. Indeed, late born calves tend to be smaller in autumn and winter than early born calves [15].

Despite the potential influence of timing of parturition on calf survival, most studies have failed to detect an influence of inter-annual variation in environmental conditions on timing of moose parturition [16, 17]; but see also [15]. In fact, although intraspecific variation in the timing of mating has been documented for some ungulate populations [1, 2, 15] the timing of mating and calving among North American moose (*Alces alces*) populations exhibits relatively low variation across >15° of latitude [16, 17].

There is ongoing debate regarding the relative importance of various selective pressures in shaping timing and synchrony of ungulate births within and among species. As part of a broader assessment of moose viability near the southern extent of their range in Ontario [18, 19, 20], we documented the seasonal and diel timing of parturition in 2 adjacent study areas. During a companion study of calf mortality, we documented a marked difference in predation rates on calves between the two areas (23.3% versus 9.0%; [21]). Here, we tested the hypothesis that predation promotes more synchronous, and earlier, birth of moose calves. Specifically, we predicted that mean date of birthing would be earlier and more synchronous (i.e. less variable) in the area receiving greater predation pressure, despite this area being subject to a later date of green up (see discussion). Additionally, although seasonal synchrony in birthing has been well documented, the recent advent of vaginal implant transmitters (VITs) containing coded signals that indicate the precise timing of expulsion (e.g. [22, 23, 24]) enabled us to objectively determine diel timing of parturition. Accordingly, we further predicted that if predation influenced the timing of parturition, proportionately more births would occur during the day-time when wolves (*Canis spp*.) and American black bears (*Ursus americanus*) tend to be less active [25, 26, 27, 28, 29].

## Materials and Methods

### Study areas

**Fig 1:**
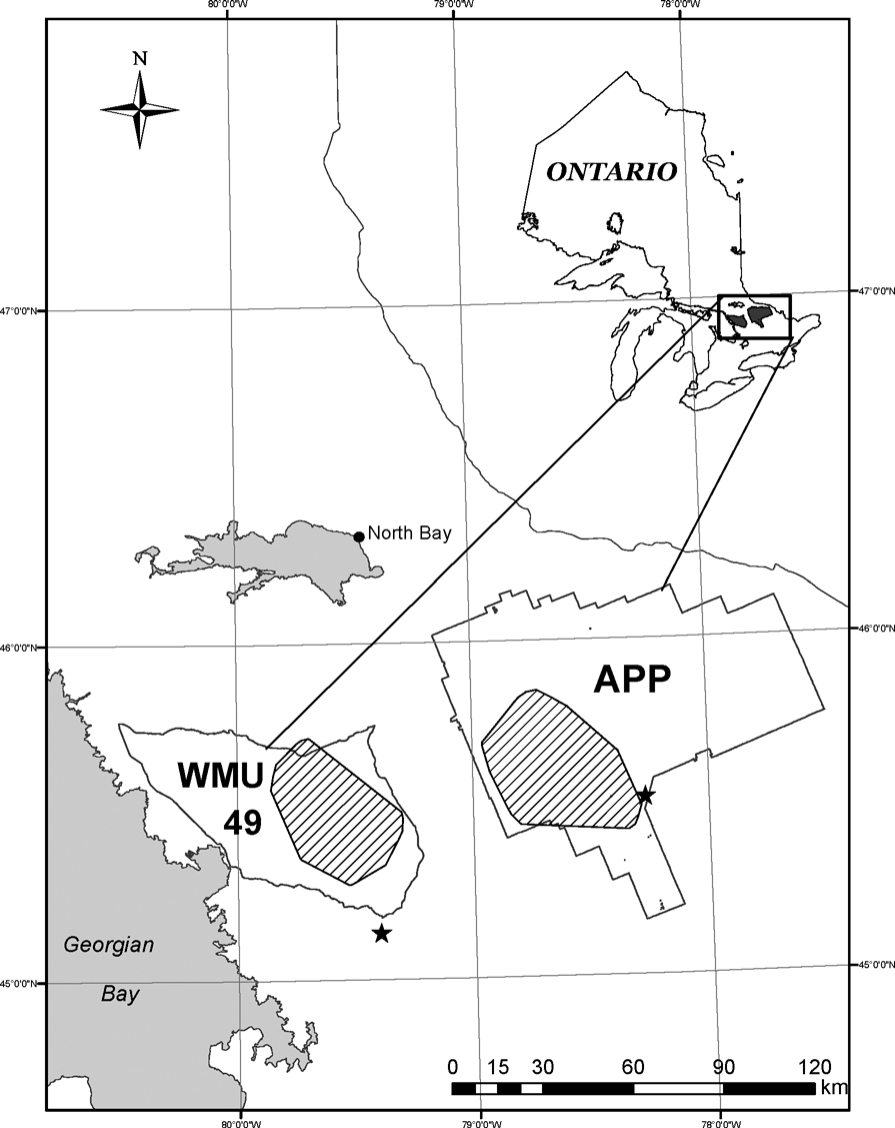
Location of study sites in Algonquin Provincial Park (APP) and Wildlife Management Unit 49 (WMU49) in central Ontario, Canada. The 2 study sites are hatched.

We studied moose in 2 nearby study sites in central Ontario, Canada (45°N, 78°W)–one in the western portion of Algonquin Provincial Park (APP) (2,000 km^2^) and the other in the southeastern portion of Wildlife Management Unit 49 (WMU49; 1,500 km^2^) (Fig 1). The overall study area was near the southern distribution of moose range in Ontario [20], and lay within the northern portion of the Great Lakes-St. Lawrence Forest Region [30]. The two study sites were separated by roughly 50 km, with western APP consisting of a protected forest with limited forest management and no moose harvest, whereas WMU49 included public and private lands where both logging and moose harvest occurred. Forest cover in APP was dominated by sugar maple (*Acer saccharum*), poplar (*Populus* spp.), American beech (*Fagus grandifolia*), yellow birch (*Betula alleghaniensis*), eastern hemlock (*Tsuga canadensis*), spruce (*Picea* spp.) and fir (*Abies* spp.); the forest composition in WMU49 was similar, although with lower abundance of hemlock and more developed and agricultural land and habitat fragmentation [31]. The study sites differed in elevation, with APP (320–580 m ASL) being approximately 200 m higher than WMU49 (73–549 m ASL). Environmental conditions during our study were typical for the area. The long term normal for daily temperatures in January and July averaged -11°C and 18°C, respectively for APP (East Gate Station: 45° 31.8’N 78° 16.2’W) and about 1°C warmer during winter and summer in WMU49 (Huntsville: 45° 20.2’N 79° 13.4’W; [32]). Potential predators of moose in the region included eastern wolves (*Canis lycaon*), eastern coyotes (C*anis latrans var*.) and American black bears. Wolves were relatively abundant in APP (2.3–3.0/ 100 km^2^ [33]) but uncommon in WMU49 where most free-ranging canids were smaller eastern coyotes and wolf-coyote hybrids [34]. Black bear densities were estimated at 37 bears/100 km^2^ (95% CI 21–66) and 32 bears/100 km^2^ (95% CI 15–57) for WMU49 and APP respectively [35].

### Calf capture

During 2007–2009 we used encapsulated vaginal implant transmitters (VITs, model M3970, Advanced Telemetry Systems, Inc Isanti, MN 55040 USA) to locate and capture calves from a random sampling of cows in both study areas. From January through March, 2007–2009 we captured 119 different adult female moose via darting or aerial net gunning and fitted them with either VHF or GPS radiocollars (see [18, 20]; Lotek 3300L, Lotek Wireless Inc., Newmarket, ON, Canada; Telemetry Solutions Quantum 5000; 5000b, Telemetry Solutions, Concord California, USA). We chemically immobilized moose in 2007 and 2008 by remote delivery of capture darts from a helicopter using a mixture of carfentanil (Wildlife Pharmaceuticals Inc., Ft. Collins, Colorado, USA) at approximately 0.0070 mg/kg combined with xylazine hydrochloride (Rompun^®^; Bayer Inc., Etobicoke, Ontario, Canada) at approximately 0.2 mg/kg [36]. We reversed this drug combination with naltrexone at approximately 0.7 mg/kg. In 2009 all captured cows were net-gunned from a helicopter (Pathfinder helicopters, Salt Lake City, Utah, USA) and manually restrained without use of chemical immobilization.

We fitted 73 of these captured adult females with VITs on 91 occasions (i.e., 18 moose were fitted with VITs during multiple years). Prior to deployment, VITs were cold sterilized for 24h in Nolvasan solution, rinsed with sterile saline, and stored individually in sterile Whirl-Pak® bags. Each VIT was placed in a sterile disposable, lubricated speculum (Animal Reproductive Systems, Chino California, USA) which was then inserted in the vagina to the cervix. VITs were held in place at the cervix by a 19 mm diameter PVC tube as the speculum was slowly pulled back. Disposable speculums were discarded, and the smaller diameter PVC tube washed with soap and water and cold sterilized in Nolvasan solution between deployments. VITs were typically shed from moose at parturition and the pulse rate changed from 40 PPM to 80 PPM when the temperature dropped below 32°C. We recovered most expelled VITS from within or immediately proximate to obvious calving beds and were only aware of two cases were VITs were expelled prior to the onset of parturition. In addition to the pulse rate change, the VITs emitted a coded pulse that enabled us to determine how long each had been shed, in increments of 30 minutes, for periods of up to 5.5 days. During the course of the study we documented a few cases where VITs had their temperature sensitive mortality switches reset by exposure to direct sunlight. VITs that had been reset by the sun were detected through repeat monitoring. For example, in rare cases we would discover that a VIT known to have been out for multiple days was suddenly sending a signal indicating more recent expulsion. Checking the timing code for VITs that had been “reset” in this manner indicated they had all been reset during the warmest part of the day and this was only ever documented for VITs shed in open meadows and exposed to direct sunlight–whereas most calving sites were in forested cover (A. McLaren and B. R. Patterson, unpublished data) where resetting of the timer was not a risk.

To avoid pseudoreplication, only a single calving date was recorded in cases where twinning was documented. All capture and handling methods followed the guidelines of the American Society of Mammalogists [37] and were approved by the Trent University Animal Care Committee as well as the Animal Care Committee of the Ontario Ministry of Natural Resources (OMNR; Permit Nos. 07–66, 08–66, 09–66). The OMNR permits were required to handle moose in both study areas; additionally we were authorized by Ontario Parks to conduct this research in Algonquin Park. No other permits or authorizations were required to conduct this research and our research did not involve endangered or protected species.

We aerially radio-tracked moose that had been fitted with VITs biweekly following capture until early-May, when daily monitoring began. At the onset of calving these moose were checked daily from the air or by a ground crew to determine calving status. When a VIT was shed, a crew of 2–4 located the calving site using a hand-held antenna and portable receiver. Calf handling and subsequent monitoring are described elsewhere [21].

### Statistical analysis

**Fig 2:**
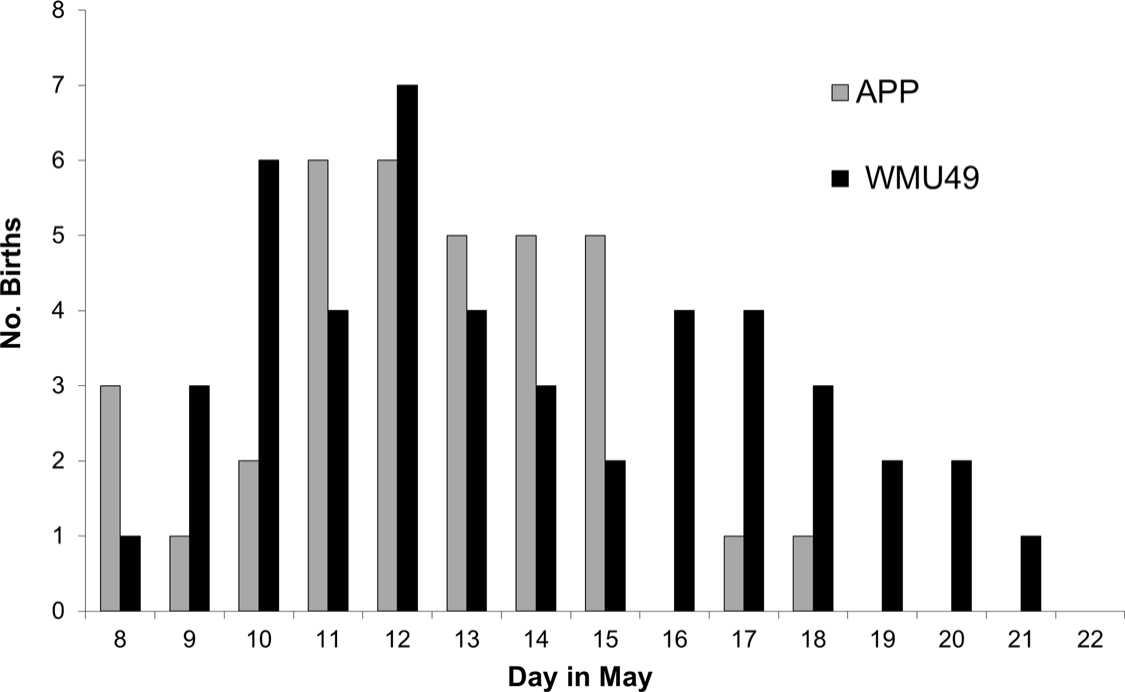
Parturition date of radio-collared adult female moose, in Algonquin Provincial Park (APP) and Wildlife Management Unit 49 (WMU49) in central Ontario, Canada, 2007–09.

We used a 2-tailed *t*-test assuming unequal variances to test whether mean calving dates differed between APP and WMU49. We used this test because it is robust to slight departures from normality [38], and the distribution of calving dates was normal in APP (*K*_*s*_ = 0.102, *P* = 0.50) and only slightly skewed in the right tail in WMU49 (*K*_*s*_ = 0.134, *P* = 0. 035; Fig 2). Although a 1-tailed test would have been more appropriate for testing the specific hypothesis that calving occurred earlier in APP owing to greater predation pressure, earlier calving in WMU49 owing to an earlier mean date of green up was also a possibility, hence the 2-tailed test. Synchrony of births is best quantified as the variance around birth date [38, 39]. Therefore, we used a 2-sample *F*-test to determine differences in variance of birth dates between the 2 areas. To test for diel patterns in the timing of parturition we used circular statistics to find the mean time of day of calving, and used Rayleigh’s z test for circular uniformity to test the null hypothesis of a uniform distribution. Small sample sizes precluded our testing for differences in diel timing of births between study areas. All Statistical testing was conducted using R (Version 2.15.3; [40]).

## Results

We successfully recorded the date of parturition of 77 calves born to cows fitted with VITs (28 in APP and 49 in WMU49; Table 1). We censored one outlying birth date in WMU49 (June 3) as this likely represented conception during second estrous and the date was > 6 standard deviations later than the mean date. In APP, an additional 44 neonatal calves were encountered without the use of VITs. However, the mean date of encountering these calves (May 17) was approximately 5 days later than the mean parturition date determined for this study area using VITs (May 12) and many of these calves were estimated to be >72 hours old upon encounter. To avoid biasing our estimate of mean parturition date in subsequent analyses we included only data from 9 calves (including 2 sets of twins, so *n* = 7 additional data points) encountered without use of VITs that we were confident were < 24 hours old at time of encounter. This determination was based published criteria [41] and experience gained while observing the calves born to VITted females at various time periods following their birth. Our observations corroborated those of Larsen et al. [41] that “.a calf < 1 day old was unable to stand, or was unsteady on its feet, or the umbilical cord was still attached, or the calf was still wet;.” In our experience a calf that was still wet or had a wet umbilical cord attached was < 12 hours old. Based on those criteria we were not only confident that each of the 9 calves in question was <24 hours old at time of discovery, but also in our ability to determine the most likely day of their birth based on their estimated age and the time of day the assessment was conducted. These additional calves increased the sample size of known parturition dates in APP to 35 (Table 1).

**Table 1:** Number of VITs shed per year during parturition by radio-collared female moose in Algonquin Provincial Park (APP) and Wildlife Management Unit 49 (WMU49), central Ontario, Canada, 2006–08.

Year	No. VITs shed during calving season	No. birthing events of VITted cows for which parturition was confirmed and timing suggested by VIT plausible	No. assumed birthing dates based on time of VIT shedding but for which timing of parturition was unconfirmed by subsequent calf observation	No. birthing events involving non-VITted cows with calves < 24 hrs. old	Total No. birthing events included in analyses
APP					
2007	5	3	1	2	6
2008	16	12	3	1	16
2009	9	7	2	4	13
	30	22	6	7	35
WMU49					
2007	13	10	1	0	11
2008	10	7	2	0	9
2009	27	25	2	0	27
	50	42	5		47

Across both study areas the first date of successful calving was May 8, with an overall mean date of calving of May 13 (Table 2). Eighty percent of calves were born by May 16 each year (Fig 2). Although small sample sizes precluded rigorous testing for inter-annual variation in parturition dates, visual inspection of the data suggested that the distribution of calving dates was similar from 2007–2009 (Table 2). From 2007–2009, mean date of calving averaged 1.5 days earlier in APP than WMU49 (*t* = -2.25, df = 81, *P* = 0.013). Calving was also more synchronous in APP, with calving dates being significantly more variable in WMU49 (variance = 12.4 versus 5.72; *F*_*34*, *47*_ = 0.463, *P* = 0.010).

**Table 2:** Number of parturition events of radio-collared female moose in Algonquin Provincial Park (APP) and Wildlife Management Unit 49 (WMU49), central Ontario, Canada, for which day of parturition was precisely known 2006–08.

		APP			WMU49	
	Mean date in May of calving	Range	*n*	Mean date in May of calving	Range	*n*
2007	May 16	13–19	3	May 14	11–20	9
2008	May 11	8–14	16	May 14	10–19	13^a^
2009	May 12	8–18	16	May 13	8–19	26
Overall	12.6	8–19	35	13.9	8–20	48

**Fig 3:**
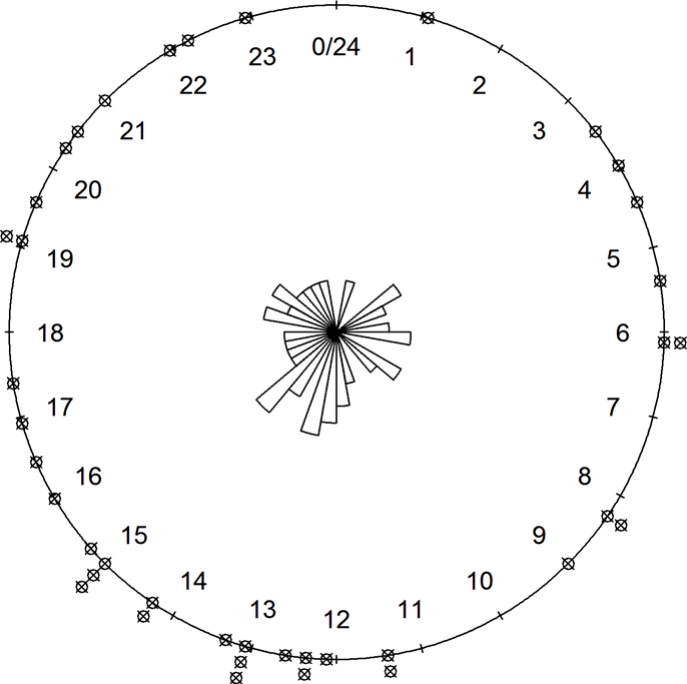
Diel time of parturition for 39 radio-collared adult female moose, central Ontario, Canada, 2007–09.

Based on the coded pulse emitted by the VITs, and excluding cases where field personal did not record the coded signal indicating time of expulsion, or where warm temperatures apparently reset the coded timer in the VITs, we recorded precise time of birth for 39 moose calves (18 and 21 in APP and WMU49, respectively). The timing of birth was not uniform throughout the 24–hr period; mean calving time was 14:49 with an angular dispersion of r = 0.299 (Z = 3.486, *P* = 0.0294; Fig 3).

## Discussion

The median parturition dates we documented (May 12 in APP (mean = 12.4), May 13 in WMU49 (mean = 13.9)) were earlier than documented in northwestern Ontario (May 14–18; [42]), Québec (May 18–June 8; [43]), or the interior of Alaska, USA (May 20–27; [10, 44, 45]). The median parturition dates we documented were also considerably earlier than the median dates of first observation of cows with neonates in part of our APP study area during 1981–84 (May 18–20; [46]). However, the observations by Addison et al. [46] were restricted to islands in APP, used by an estimated <20% of calving moose in the park, and first observation of cows with calves may not accurately reflect the actual day of parturition, as suggested by the portion of our data on age of captured moose calves for which we used similar capture techniques as Addison et al. [46]. We suggest that the use of VITs deployed in a random sample of cows in winter provided a less biased measure of parturition dates for moose in our study areas.

We also observed later and less synchronous parturition of moose calves in the study area with lighter predation pressure (WMU49) than observed in nearby APP which, at only 50 km to the east, had similar habitat and climatic features. Wolves and black bears were the only significant predators of moose during this study. Our assertion that predation pressure was lighter in WMU49, despite potentially higher bear densities there, was based primarily on the results of a companion study of calf mortality [21]; however, other lines of evidence also suggest this was the case. Wolves were relatively abundant in APP, and although wolf-like canids were likely more abundant in WMU49, most free-ranging canids in the area were smaller eastern coyotes and wolf-coyote hybrids [34] that are less likely to prey on moose calves (e.g. [47]).

Among black bears, large males may be more likely to prey on ungulates [48, 49], including moose calves [44], and although bear densities were similar between areas the population in WMU49 was hunted. Harvest of bears across Ontario is skewed towards males, especially subadult males [50]. Therefore, hunting in WMU49 may have altered both the sex and age ratios of the bear population relative to that in APP such that there were fewer large male bears capable of successfully preying on moose calves in the area. Adult female black bears do prey on moose calves, but such predation attempts are potentially risky and a defensive cow moose can be dangerous prey for much smaller adult female black bears [51]. Such predation attempts are likely less risky for the much larger adult male black bears, and adult male black bears can even prey successfully on adult cow moose [52], so a black bear population with a lower proportion of adult males would be expected to exert less predation pressure on moose.

That predation pressure differed between the two areas was also consistent with the marked differences in defensive behavior exhibited by cows in the 2 study areas. Maternal defense of calves was almost absent in WMU49, with cows generally retreating when they first observed our approach to their calf [21]. In APP, maternal defense became increasingly common during the course of our study, to the extent that by 2008 we were unable to handle the calves of cows without first chemically immobilizing the cows because of strong aggression [21]. Although the lack of maternal defensive behavior in WMU49 may also reflect a learned flight response to hunting by humans occurring in this study area but not APP, hunted moose in other jurisdictions experiencing significant predation on calves exhibit similarly aggressive defense of their calves (e.g., [53]) suggesting that higher rates of predation on calves in APP was a more likely cause of this defensive behavior.

Algonquin Provincial Park was established as a protected area in 1893. However, given that wolves were lethally controlled in APP until 1958 [54] it is not clear how long predation pressure by wolves on moose calves has been appreciably different between the 2 study areas. Regardless, it may seem difficult to imagine measurable shifts in the timing and synchrony of births by moose in such a short period of time (i.e., since the establishment of APP as a protected area in 1893, or since the establishment of wolf protection in APP in 1958). However, moose captured in the Northern Hemisphere and introduced to New Zealand quickly adjusted their timing of births to occur in spring in New Zealand, which was 6 months different from their capture location [55]. Similarly, bighorn sheep (*Ovis canadensis*) captured from established populations in Utah, Alberta, Montana, and Colorado and translocated into two separate populations in northern Utah adjusted timing and synchrony of parturition to the environmental conditions of their release sites within 5 years of reintroduction [39].

The mean calving date in APP was only 1.5 days earlier than in WMU49, and similar to the discussion above, some might find it difficult to envision such a subtle difference in parturition date conferring much of a selective advantage in terms of predator avoidance. However, calves during our study gained mobility quickly after birth and by ca. 3 days of age were often difficult to capture by hand on the ground [21]. Furthermore, in an area of south-central Alaska that received heavy predation pressure, moose calves that survived to autumn (n = 86) were born an average of 1.8 days earlier than the mean calving date for the overall population (n = 248 calves [9]). As discussed by Testa [9], the selective force of predation on birth dates over generations is expected to be cumulative and the optimum birth date in any given year is always earlier than that actually occurring. Over time, the selective pressure of predation should push heritable variation in timing of parturition to the earliest possible date until balanced out by opposing selective forces (e.g. timing of green-up [9, 10]).

Bowyer et al. [10] noted that extirpation of wolves and grizzly bears (*Ursus arctos*) from much of their original distribution in North America has produced a patchy distribution in the primary predators of moose across the continent. They suggested that the continental synchrony in onset and duration of parturition among North American moose, despite spatially variable predation pressure, indicates moose are tracking long-term patterns of climate to time reproduction [10]. However, the observed differences in timing and synchrony of parturition observed between our 2 study areas were not likely due to differences in climatic conditions or plant phenology because temperatures were actually slightly warmer in WMU49 [32, 56]. Further, between 2007 and 2009, average green-up date and peak NDVI date occurred two days earlier in WMU49 compared to APP (13 April and 15 April, and 3 and 5 July, respectively [57]; yet moose calved later and less synchronously in WMU49. We also discount differences in the genetic composition or nutritional plane of moose between the 2 study areas as an explanation for some or all of the differences in parturition we observed. Although the genetic composition of a population has been implicated as a driver for timing and synchrony of births in reintroduced bighorn sheep [58], moose in our two study areas exhibited no genetic differences [59]. Pregnancy rates, blood-based condition indices [20], twinning rates [21], and weights of calves <36 hours old at time of handling (APP = 15.5 kg, *n* = 18; WMU49 = 14.9 kg, *n* = 30; t _18, 30_ = 0.880, *P* = 0.383) were all similar between areas indicating no apparent differences in condition of females between areas.

**Fig 4:**
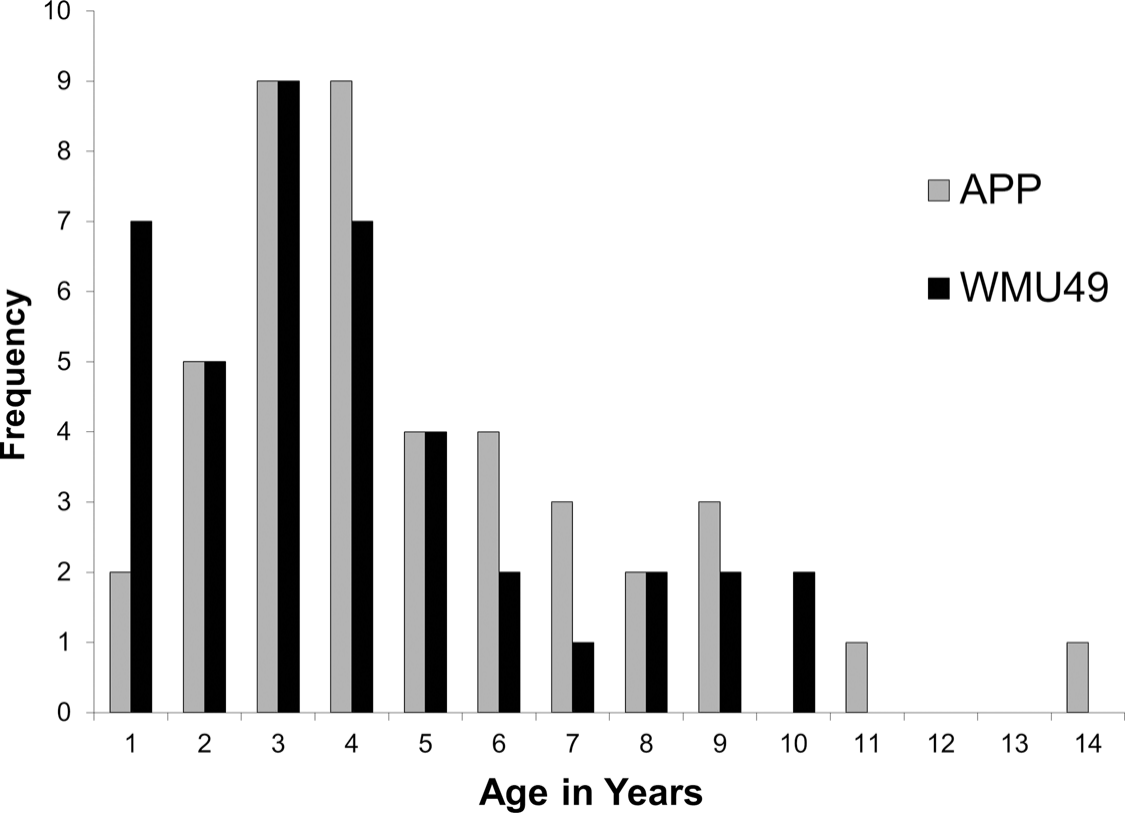
Age distributions of radio-collared female moose in Algonquin Provincial Park (APP) and Wildlife Management Unit 49 (WMU49), central Ontario, Canada, 2006–07.

Another alternative hypothesis warranting consideration relates to maternal age. On the island of Vega in northern Norway, younger female moose gave birth later than older females [15, 60]. Based on analysis of cementum annuli in extracted vestigial teeth of cow moose captured during our study (*n* = 84) the mean age of cows from APP was 4.8 ± 0.42 versus 4.0 ± 0.40 years in WMU49 ([20]; Fig 4). However, the difference in age distributions between the 2 areas was largely a result of substantially more yearling cows in WMU49 (Fig 4), probably owing to the higher calf survival documented for this area [21]. Also, all of the known yearling female moose radiocollared during this study were captured in winter 2006 (7 in APP, 2 in WMU49), a year before the present study began, meaning all female radiocollared moose recruited into the study in 2007 would have been at least 2 years old. Beginning in 2007 we explicitly instructed capture crews to focus on mature female moose, and none of 15 newly captured and aged cows in 2007 (8 and 7 in APP and WMU49, respectively) were yearlings. We did not section teeth for aging in 2008 or 2009 but based on crew instruction and pictures of the captures we believe few if any yearlings were included in the samples of parturient cows during these years. Regardless, given that differences in birthing dates with age are usually driven by the first few primiparous age classes (>2 years old for moose; [15, 60, 61]), and that the distribution of these age classes was essentially the same between the two study areas even when the 2006 captures are considered (Fig 4), we suggest there were insufficient differences in age structure between the two study areas to drive the earlier parturition dates in APP.

### Diel timing of births

Although birth dates are well documented for many ungulate species, given the recent advent of VITs, there are few data on precise diel timing of births. Small sample sizes precluded our testing for differences in diel timing of births between study areas, but in pooling study areas we documented a disproportionate number of births in the afternoon. Among mammals the onset of labour is immediately preceded by a sharp increase in free circulating oestrogens [62]. The proximate triggers of this increase in free circulating oestrogens are not definitively known, but cortisol is also involved in the process and Nathanielsz [63] suggests that animals tend to give birth when they feel the most secure and comfortable. Changes in plasma cortisol levels likely represent the link between stress related to perceived predation risk and the cascading hormonal changes that may influence the onset of labour [62, 63].

Throughout much of the year wolves tend to be less active during mid-day and most active during crepuscular and dark periods [26, 27, 29]. Though diel activity patterns of black bears are more variable across their range, they often have reduced activity levels during the mid-day period and a spike in activity in early evening [25, 28]. Thus moose may be less stressed and more likely to enter labor at the time of day when they are more likely to be resting and least likely to be discovered by wolves or bears. We note however that our data provide only weak support for this hypothesis given that our pooled sample of birth times contained slightly more data from WMU49 where predation pressure was relatively light. This suggests either that several factors are influencing this trait and/or that there is not strong selection against having calves during the afternoon such that the trait persists even in areas with relatively light predation pressure. Nonetheless, predation risk clearly influences other aspects of moose life history [64, 65] and Addison et al. [66] previously suggested that predator avoidance likely exerted a strong influence on birthing site selection by moose in APP. Accordingly, it seems reasonable that predation risk may also influence the diel timing of parturition.

Alternatively, the disproportionate number of afternoon births may simply reflect selection for birthing during the warmest time of day, as temperatures occasionally reached freezing over night during calving in this study and neonatal calves can be sensitive to exposure during harsh weather [67, 68]. However, Bowyer et al. [10] were dismissive of the temperature hypothesis based on the large body size of moose calves relative to other smaller-bodied ungulates born under similar conditions. Indeed, non-predation-related mortalities are rare among neonatal moose calves in Alaska and the Yukon [41, 69]. Finally, a disproportionate number of afternoon births may simply reflect the fact that moose themselves tend to be less active at this time of day, potentially favouring the onset of labour [62, 63]. Additional investigations of calving by captive and free-ranging moose are required to clarify the potential influence of predation risk in driving the seasonal and diel timing of parturition.

## Supporting Information

S1 Table Date and time of day of parturition for moose calves in central Ontario Canada, 2007–2009. (DOCX)

## References

[1] Bronson EH (1989) Mammalian reproductive biology The University of Chicago Press, Chicago, Illinois, 325 pp.

[2] Bunnell FL (1982) The lambing period of mountain sheep: synthesis, hypotheses, and tests. Canadian Journal of Zoology 60: 1–14.

[3] Edwards RY, Ritcey RW (1958) Reproduction in a moose population. Journal of Wildlife Management 22: 261–268.

[4] Estes RD (1976) The significance of breeding synchrony in the wildebeest. East African Wildlife Journal 14: 135–152.

[5] Schwartz CC, Hundertmark KJ (1993) Reproductive characteristics of Alaskan moose. The Journal of Wildlife Management 57: 454–468.

[6] Bergerud AT (1975) The reproductive season of Newfoundland caribou. Canadian Journal of Zoology 53: 1213–1221. 1146 10.1139/z75-145

[7] Estes DR, Estes RK (1979) The birth and survival of wildebeest calves. Zeitschrift ffir Tierpsychologie 50: 45–95. 10.1111/j.1439-0310.1979.tb01015.x 494841

[8] Berger J (1992) Facilitation of reproductive synchrony by gestation adjustment in gregarious mammals: a new hypothesis. Ecology 73: 323–329.

[9] Testa JW (2002) Does predation on neonates inherently select for earlier births? Journal of Mammalogy 83: 699–706.

[10] Bowyer RT, Van Ballenberghe V, Kie JG (1998) Timing and synchrony of parturition in Alaskan moose: long term verses proximal effects of climate. Journal of Mammalogy 79: 1332–1344.

[11] Festa-Bianchet M (1988) Birthdate and survival in bighorn lambs (*Ovis canadensis*). Journal of Zoology (London) 214: 653–661.

[12] Rutberg AT (1987) Adaptive hypotheses of birth synchrony in ruminants: an interspecific test. The American Naturalist 130: 692–710.

[13] Adams LG, Singer FJ, Dale BW (1995) Caribou calf mortality in Denali National Park. Alaska. Journal of Wildlife Management 59: 584–594.

[14] Keech MA, Bowyer RT, Ver Hoef JM, Boertje RD, Dale BW, Stephenson TR. (2000) Life-history consequences of maternal condition in Alaskan moose. Journal of Wildlife Management 64: 450–462.

[15] Solberg EJ, Heim M, Grøtan V, Sæther BE, Garel M (2007) Annual variation in maternal age and calving date generate cohort effects in moose (*Alces alces*) body mass. Oecologia 154:259–271. 17713790 10.1007/s00442-007-0833-9

[16] Schwartz CC (1998) Reproduction, natality, and growth Pages 141–171, in Ecology and management of the North American moose (Franzmann A. W. and Schwartz C. C., eds.), Smithsonian Institution Press, Washington, D.C., 733 pp.

[17] Sigouin D, Ouellet JP, Courtois R (1997) Geographical variations in the mating and calving periods of moose. Alces 33: 85–95.

[18] Lowe SJ, Patterson BR, Schaefer J (2010) Lack of behavioral responses of moose (*Alces alces*) to high ambient temperatures near the southern periphery of their range. Canadian Journal of Zoology 88: 1032–1041.

[19] McLoughlin PD, Vander Wal E, Lowe SJ, Patterson BR, Murray DL (2011) Seasonal shifts in habitat selection of a large herbivore and the influence of human activity. Basic and Applied Ecology 12: 654–663.

[20] Murray DL, Hussey K, Finnegan L, Lowe SJ, Price G, Benson J, et al (2012) Assessment of the status and viability of a moose population at its southern range limit in Ontario. Canadian Journal of Zoology 90: 422–434.

[21] Patterson BR, Benson JF, Middel KR, Mills KJ, Obbard ME (2013) Moose calf mortality in central Ontario, Canada. Journal of Wildlife Management 77:832–841.

[22] Bishop CJ, Freddy DJ, White GC, Watkins BE, Stephenson TR, Wolfe LL. (2007) Using vaginal implant transmitters to aid in capture of mule deer neonates. Journal of Wildlife Management 71: 945–954.

[23] Bowman JL, Jacobson HA (1998) An improved vaginal implant transmitter for locating white-tailed deer birth sites and fawns. Wildlife Society Bulletin 26: 295–298.

[24] Johnstone-Yellin T L, Shipley LA, Myers WL (2006) Effectiveness of vaginal implant transmitters for locating neonatal mule deer fawns. Wildlife Society Bulletin 34: 338–344.

[25] Bridges AS, Vaughan MR, and Klenzendorf SA (2004) Seasonal variation in American black bear *Ursus americanus* activity patterns: quantification via remote photography. Wildlife Biology 10: 277–284.

[26] Eriksen A, Wabakken P, Zimmermann B, Andreassen HP, Arnemo JM, Gundersen H, et al (2011) Activity patterns of predator and prey: a simultaneous study of GPS-collared wolves and moose. Animal Behaviour 81: 423–431.

[27] Fuller TK (1991) Effect of snow depth on wolf activity and prey selection in north central Minnesota. Canadian Journal of Zoology 69: 283–287.

[28] Larivière S, Huot J, Samson C (1994) Daily activity patterns of female black bears in a northern mixed-forest environment. Journal of Mammalogy 75: 613–620.

[29] Theuerkauf J, Jedrzejewski W, Schmidt K, Okarma H, Ruczynski I, Sniezko S, et al (2003) Daily patterns and duration of wolf activity in the Bialowieza Forest, Poland. Journal of Mammalogy 84: 243–253.

[30] Rowe JS (1972) Forest Regions of Canada. Canadian Forest Service Publication No. 1300. Department of Fisheries and the Environment, Ottawa, Ontario, Canada.

[31] Crins WJ, Gray PA, Uhlig PWC, Wester M (2008) The ecosystems of Ontario, part 2: Ecodistricts Ontario Ministry of Natural Resources, Peterborough, Ontario, Canada.

[32] Environment Canada (2008) Climate data online. National Climate Data and Information Archive [online]. Available: http://climate.weatheroffice.ec.gc.ca/climateData/canada_e.html [accessed on 5 January 2009].

[33] Patterson BR, Quinn NWS, Becker EF, Meier DB (2004) Estimating wolf densities in forested areas using network sampling of tracks in snow. Wildlife Society Bulletin 32: 938–947.

[34] Benson J, Patterson BR, Wheeldon TJ (2012) Spatial genetic and morphologic structure of wolves and coyotes in relation to environmental heterogeneity in a Canis hybrid zone. Molecular Ecology 21: 5934–5954. 10.1111/mec.12045 23173981

[35] Howe EJ, Obbard ME, Kyle CJ (2013) Combining data from 43 standardized surveys to estimate densities of female American black bears by spatially explicit capture–recapture. Population Ecology 55: 595–607.

[36] Canadian Association of Zoo and Wildlife Veterinarians (2009) The chemical immobilization of wildlife, 3rd ed. Canadian Association of Zoo and Wildlife Veterinarians Winnipeg, Manitoba, Canada.

[37] Sikes RS, Gannon WL, the Animal Care and Use Committee of the American Society of Mammalogists (2011) Guidelines of the American Society of Mammalogists for the use of wild mammals in research. Journal of Mammalogy 92: 235–253. 10.1093/jmammal/gyw078 PMC5909806 29692469

[38] Johnson DS, Barry RP, Bowyer RT (2004) Estimating timing of life-history events with coarse data. Journal of Mammalogy 85: 932–939.

[39] Whiting JC, Bowyer RT, Flinders JT, Egget DL (2011) Reintroduced bighorn sheep: fitness consequences of adjusting parturition to local environments. Journal of Mammalogy: 92: 213–220.

[40] R Development Core Team (2011) R: a language and environment for statistical computing R Foundation for Statistical Computing, Vienna, Austria.

[41] Larsen DG, Gauthier DA, Markel RL (1989) Causes and rate of moose mortality in the southwest Yukon. Journal of Wildlife Management 53: 548–557.

[42] Welch ID, Rodgers AR, McKinley RS (2000) Timber harvest and calving site fidelity of moose in northwestern Ontario. Alces 36: 93–103.

[43] Laurian C, Ouellet JP, Courtois R, Breton L, St-Onge S (2000) Effects of intensive harvesting on moose reproduction. Journal of Applied Ecology 37: 515–531.

[44] Bertram MR, Vivion MT (2002) Moose mortality in eastern interior Alaska. Journal of Wildlife Management 66: 747–756.

[45] Testa JW, Becker EF, Lee GR (2000) Temporal patterns in the survival of twin and single moose (*Alces Alces*) calves in Southcentral Alaska. Journal of Mammalogy 81: 162–168.

[46] Addison EM, McLaughlin RF, Fraser DJH, Buss ME (1993) Observations of pre- and post-partum behaviour of moose in central Ontario. Alces 29: 27–33.

[47] Benson JF, Patterson BR (2013) Moose (*Alces alces*) predation by eastern coyotes (*Canis latrans*) and eastern coyote × eastern wolf (*Canis latrans* × *Canis lycaon*) hybrids. Canadian Journal of Zoology 91: 1–5.

[48] Gunther KA, Renkin RA (1990) Grizzly bear predation on elk calves and other fauna of Yellowstone National Park. International Conference on Bear Research and Management 8: 329–334.

[49] Jacoby ME, Hilderbrand GV, Servheen C, Schwartz CC, Arthur SM, Hanley TA, et al (1999) Trophic relations of brown and black bears in several western North American ecosystems. Journal of Wildlife Management 63: 921–929.

[50] McLaren M, Dix-Gibson L, Armstrong T, Dawson N, Obbard ME, Landriault L, et al. (2009) An approach to assessment of harvested black bear populations in Ontario. Southern Science and Information Section Technical Report, No. 127. Ontario Ministry of Natural Resources, Southern Science and Information Section, Bracebridge, Ontario, Canada.

[51] Obbard ME, Campbell GD, Schenk A (2000) Evidence for fatal injury inflicted on a female black bear by a moose. Northeast Wildlife 55:57–62.

[52] Austin MA, Obbard ME, Kolenosky GB (1994) Evidence for a black bear, *Ursus americanus*, killing an adult moose, *Alces alces*. Canadian Field-Naturalist 108: 236–238.

[53] Ballard WB, Franzmann AW, Taylor KP, Spraker TH, Schwartz CC, Peterson RO. (1979) Comparison of techniques utilized to determine moose calf mortality in Alaska. Proceedings from the North American Moose Conference and Workshop 15: 362–387.

[54] Pimlott DH, Shannon JA, Kolenosky GB (1969) The ecology of the timber wolf in Algonquin Provincial Park. Ontario Department of Lands and Forests, Fish and Wildlife Research report No. 87, 92 pp.

[55] Marshall FHA, Cambridge FRS (1937) On the changeover in the oestrous cycle in animals after transference across the equator, with further observations on the incidence of the breeding seasons and the factors controlling sexual periodicity. Proceedings of the Royal Society of London, B. Biological Sciences 122: 413–428.

[56] Quinn NWS (2005) Reconstructing changes in abundance of White-tailed Deer, *Odocoileus virginianus*, Moose, *Alces alces*, and Beaver, *Castor canadensis*, in Algonquin Park, Ontario, 1860–2004. Canadian Field-Naturalist 119: 330–342.

[57] United States Geological Survey EROS Centre (2014) USGS Conterminous U.S. 1 km AVHRR Remote Sensing Phenology Data [online]. Available: http://phenology.cr.usgs.gov/ [accessed May 27, 2014].

[58] Hogg JT, Forbes SH, Steele BM, Luikart G (2006) Genetic rescue of an insular population of large mammals. Proceedings of the Royal Society of London, B. Biological Sciences 273: 1491–1499. 10.1098/rspb.2006.3477 PMC1560318 16777743

[59] Finnegan LA, Wilson PJ, Price GN, Lowe SJ, Patterson BR, Fortin MJ, et al (2012) The complimentary role of genetic and ecological data in understanding population structure: A case study using moose (*Alces alces*). European Journal of Wildlife Research 58: 415–423.

[60] Sæther BE, Solberg EJ, Heim M (2003) Effects of altering sex ratio structure on the demography of an isolated moose population. The Journal of Wildlife Management 67: 455–466.

[61] Boer AH (1992) Fecundity of North American moose (*Alces alces*): a review. Alces supplement 1 (1992): 1–10.

[62] Rawlings NC, Ward WR (1976) Changes in steroid homrones in plasma and myometrium and uterine activity in ewes during late pregnancy and parturition. Journal of reproductive fertility 48: 355–360. 10.1530/jrf.0.0480355 994107

[63] Nathanielsz PW (1998) Comparative studies on the initiation of labor. European Journal of Obstetrics & Gynecology and Reproductive Biology 78: 127–132. 9622309 10.1016/s0301-2115(98)00058-x

[64] Berger J (2007) Fear, human shields and the redistribution of prey and predators in protected areas. Biology Letters 3: 620–623. 17925272 10.1098/rsbl.2007.0415 PMC2391231

[65] White KS, Testa JW, Berger J (2001) Behavioral and ecological effects of differential predation pressure on moose in Alaska. Journal of Mammalogy 82: 422–429.

[66] Addison EM, Smith JD, McLaughlin RF, Fraser DJH, Joachim DG (1990) Calving sites of moose in central Ontario. Alces 26: 142–153.

[67] Griffin KA, Hebblewhite M, Robinson HS, Zager P, Barber-Meyer S, Christianson D, et al (2011) Neonatal mortality of elk driven by climate, predator phenology and predator community composition. Journal of Animal Ecology 80: 1246–57. 10.1111/j.1365-2656.2011.01856.x 21615401

[68] Hegel TM, Mysterud A, Ergon T, Loe LE, Huettmann F, et al (2010) Seasonal effects of Pacific-based climate on recruitment in a predator-limited large herbivore. Journal of Animal Ecology 79: 471–482. 10.1111/j.1365-2656.2009.01647.x 20002863

[69] Keech MA, Lindberg MS, Boertje RD, Valkenburg P, Taras B, Boudreau TA, et al (2011) Effects of predator treatments, individual traits, and environment on moose survival in Alaska. Journal of Wildlife Management 75: 1361–1380.

