# Supplementary material for: Does Predation Influence the Seasonal and Diel Timing of Moose Calving in Central Ontario, Canada?
Source: PLoS One. 2016 Apr 15;11(4):e0150730. doi: 10.1371/journal.pone.0150730 (PMC4833312; doi:10.1371/journal.pone.0150730)
Supplement: S1 Table — (DOCX) [file pone.0150730.s001.docx]

| **Study Area** | **Date** | **Time of day (24 hr)** |
| --- | --- | --- |
| APP | 13-May-07 |  |
| APP | 16-May-07 |  |
| APP | 19-May-07 | 13:00 |
| APP | 08-May-08 |  |
| APP | 10-May-08 | 14:30 |
| APP | 11-May-08 | 12:40 |
| APP | 11-May-08 | 13:21 |
| APP | 11-May-08 | 15:30 |
| APP | 11-May-08 | 20:20 |
| APP | 12-May-08 | 15:11 |
| APP | 12-May-08 | 16:00 |
| APP | 12-May-08 |  |
| APP | 12-May-08 |  |
| APP | 13-May-08 | 23:00 |
| APP | 13-May-08 |  |
| APP | 13-May-08 |  |
| APP | 13-May-08 |  |
| APP | 14-May-08 | 22:00 |
| APP | 14-May-08 |  |
| APP | 08-May-09 | 13:00 |
| APP | 08-May-09 | 13:15 |
| APP | 09-May-09 |  |
| APP | 10-May-09 | 21:00 |
| APP | 11-May-09 | 8:30 |
| APP | 12-May-09 |  |
| APP | 12-May-09 |  |
| APP | 13-May-09 | 19:00 |
| APP | 14-May-09 | 19:45 |
| APP | 14-May-09 |  |
| APP | 14-May-09 |  |
| APP | 15-May-09 | 5:35 |
| APP | 15-May-09 |  |
| APP | 15-May-09 |  |
| APP | 15-May-09 |  |
| APP | 18-May-09 | 12:30 |
| WMU49 | 11-May-07 | 6:10 |
| WMU49 | 11-May-07 |  |
| WMU49 | 12-May-07 |  |
| WMU49 | 12-May-07 |  |
| WMU49 | 13-May-07 |  |
| WMU49 | 16-May-07 |  |
| WMU49 | 19-May-07 |  |
| WMU49 | 20-May-07 |  |
| WMU49 | 20-May-07 |  |
| WMU49 | 10-May-08 | 4:00 |
| WMU49 | 10-May-08 | 6:10 |
| WMU49 | 10-May-08 | 11:49 |
| WMU49 | 12-May-08 | 1:00 |
| WMU49 | 13-May-08 | 12:50 |
| WMU49 | 15-May-08 | 14:30 |
| WMU49 | 16-May-08 |  |
| WMU49 | 16-May-08 |  |
| WMU49 | 17-May-08 | 4:50 |
| WMU49 | 17-May-08 |  |
| WMU49 | 18-May-08 |  |
| WMU49 | 18-May-08 |  |
| WMU49 | 19-May-08 |  |
| WMU49 | 03-Jun-08 |  |
| WMU49 | 08-May-09 | 8:20 |
| WMU49 | 09-May-09 | 15:00 |
| WMU49 | 09-May-09 | 22:30 |
| WMU49 | 09-May-09 |  |
| WMU49 | 10-May-09 | 19:00 |
| WMU49 | 10-May-09 |  |
| WMU49 | 10-May-09 |  |
| WMU49 | 11-May-09 |  |
| WMU49 | 11-May-09 |  |
| WMU49 | 12-May-09 | 17:00 |
| WMU49 | 12-May-09 |  |
| WMU49 | 12-May-09 |  |
| WMU49 | 12-May-09 |  |
| WMU49 | 13-May-09 | 15:00 |
| WMU49 | 13-May-09 |  |
| WMU49 | 14-May-09 | 9:00 |
| WMU49 | 14-May-09 | 17:45 |
| WMU49 | 14-May-09 | 20:45 |
| WMU49 | 15-May-09 | 12:00 |
| WMU49 | 16-May-09 |  |
| WMU49 | 17-May-09 | 3:45 |
| WMU49 | 17-May-09 |  |
| WMU49 | 17-May-09 |  |
| WMU49 | 18-May-09 |  |
| WMU49 | 19-May-09 | 11:30 |
| WMU49 | 21-May-09 | 16:40 |
